# Transporter hypothesis in pharmacoresistant epilepsies. Is it at the central or peripheral level?

**DOI:** 10.1002/epi4.12537

**Published:** 2021-10-29

**Authors:** Liliana Czornyj, Jerónimo Auzmendi, Alberto Lazarowski

**Affiliations:** ^1^ Neurology Service “Juan P. Garrahan” National Children's Hospital Buenos Aires Argentina; ^2^ Institute for Research in Physiopathology and Clinical Biochemistry (INFIBIOC) Clinical Biochemistry Department School of Pharmacy and Biochemistry University of Buenos Aires Buenos Aires Argentina; ^3^ National Council for Scientific and Technical Research (CONICET) Buenos Aires Argentina

**Keywords:** central action, membrane depolarization, peripheral action, refractory epilepsy, sudden unexpected death in epilepsy (SUDEP), transporter hypothesis

## Abstract

The multidrug‐resistance (MDR) phenotype is typically observed in patients with refractory epilepsy (RE) whose seizures are not controlled despite receiving several combinations of more than two antiseizure medications (ASMs) directed against different ion channels or neurotransmitter receptors. Since the use of bromide in 1860, more than 20 ASMs have been developed; however, historically ~30% of cases of RE with MDR phenotype remains unchanged. Irrespective of metabolic biotransformation, the biodistribution of ASMs and their metabolites depends on the functional expression of some ATP‐binding cassette transporters (ABC‐t) in different organs, such as the blood‐brain barrier (BBB), bowel, liver, and kidney, among others. ABC‐t, such as P‐glycoprotein (P‐gp), multidrug resistance–associated protein (MRP‐1), and breast cancer–resistance protein (BCRP), are mainly expressed in excretory organs and play a critical role in the pharmacokinetics (PK) of all drugs. The *transporter hypothesis* can explain pharmacoresistance to a broad spectrum of ASMs, even when administered simultaneously. Since ABC‐t expression can be induced by hypoxia, inflammation, or seizures, a high frequency of uncontrolled seizures increases the risk of RE. These stimuli can induce ABC‐t expression in excretory organs and in previously non‐expressing (electrically responsive) cells, such as neurons or cardiomyocytes. In this regard, an alternative mechanism to the classical pumping function of P‐gp indicates that P‐gp activity can also produce a significant reduction in resting membrane potential (ΔΨ0 = −60 to −10 mV). P‐gp expression in neurons and cardiomyocytes can produce membrane depolarization and participate in epileptogenesis, heart failure, and sudden unexpected death in epilepsy. On this basis, ABC‐t play a peripheral role in controlling the PK of ASMs and their access to the brain and act at a central level, favoring neuronal depolarization by mechanisms independent of ion channels or neurotransmitters that current ASMs cannot control.

**Key points**

- The 30% of MDR‐phenotype in RE is constant, suggesting that it is independent to the specific action of each ASMs currently in use.
- Given the mentioned properties, all the drugs described in the pharmacopeia can be substrates of, at least, one of these transporters.
- The inducible characteristics and the genetic polymorphisms of ABC‐t, justify strong interindividual differences in therapeutic responses.
- The ability of P‐gp to depolarize neuronal membranes and cardiomyocytes could be associated with epileptogenesis, and SUDEP, respectively.

## 1 INTRODUCTION

Fifty percent of newly diagnosed epilepsy patients obtain complete seizure control with the first antiseizure medication (ASM) trial, and 13% more enter remission with the addition of a second drug. However, despite different combinations of ASMs, 30%–40% of the remaining patients will not obtain satisfactory seizure control. Although the wrong choice of ASMs may be the cause of treatment failure, this is not drug resistance.

With the correct therapeutic scheme, S. M. Sisodiya (2006) proposed that patients considered drug‐resistant might not remain so as newer ASMs are developed or designed to target previously unappreciated pathophysiological mechanisms. Consequently, individuals considered to have "drug‐resistant” epilepsy may not be so; the appropriate drugs for treatment are not yet available.^1^

**FIGURE 1:**
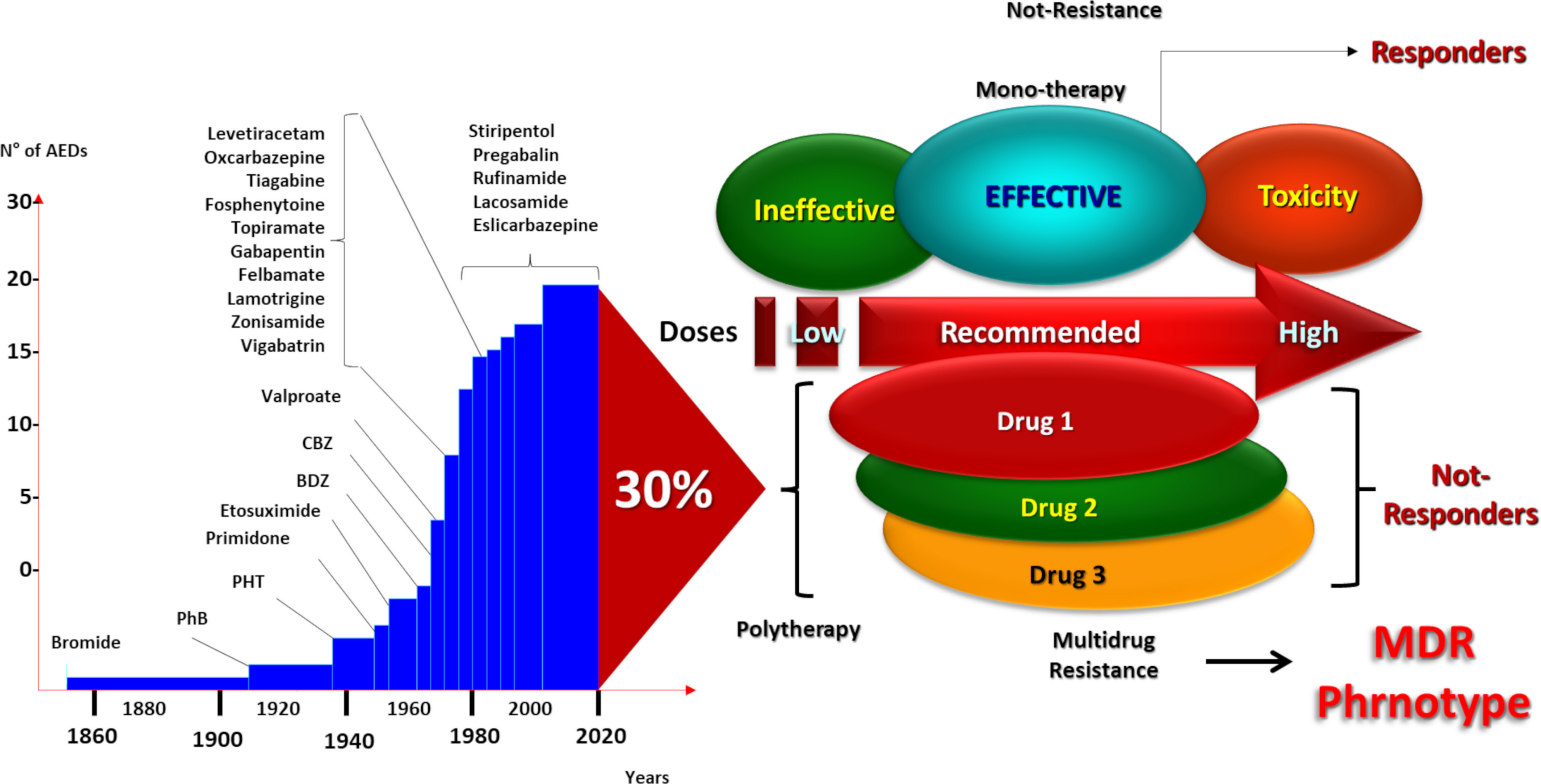
Over time, antiseizure medications have been able to control seizures in most cases. However, without exception, all ASMs fail in 30% of cases, even when combination therapy is administered. This observation suggests that, once the MDR phenotype is established, the seizure‐generating mechanism is no longer the target of the ASM of choice. ASMs: antiseizure medications; MDR: multidrug resistance

Since the first ASM, bromide, in 1860, the development of new ASMs has continued to date, and more than 20 different compounds have been designed and included for clinical settings (Figure 1). During the process of drug discovery, a new compound or molecule found could be more effective, with high affinity and specificity on a desired target in many cases. However, the clinical application of this ideal compound will be significantly influenced by different mechanisms driving its absorption, biodistribution, metabolism, excretion, and central nervous system (CNS) penetration rate.

The clinical multidrug‐resistance (MDR) phenotype, commonly observed in patients with RE, indicates that once it is settled, whatever the combination of ASMs used, a high percentage of cases will still remain with no seizure control. As this percentage has remained unchanged over time, new drugs designed against different targets destined to suppress the mechanisms that drive the MDR‐phenotype are an urgent necessity.

We think that the *transporter hypothesis* brings together biological and pharmacological factors that may explain the clinical phenomenon of MDR in refractory epilepsy (RE). It is important to highlight that “the transporters hypothesis” is based on the increased expression of some ABC‐transporters (ABC‐t) as P‐glycoprotein (P‐gp), BCRP and MRPs, mainly at the level of the vascular endothelial cells (VECs) and cellular elements of the BBB.^2^, ^3^

In the development of this critical review, we will give evidence of the role of ABC‐t in the pharamacokinetic alteration ASMs, which we consider as a peripheral mechanism. On the other hand, we indicate the potential epileptogenic role of ABC‐t, when they are expressed in the neurons, and assuming this as a central mechanism.

After V. Ling discovered that the induction of P‐gp expression in tumor cell membrane conferred resistance to drugs that had never been presented to those cells,^4^, ^5^ the entire engineering of cancer treatment changed in search of new drugs capable of overcoming this mechanism, leading to the emergence of the so‐called precision medicine. This concept becomes even more critical when trying to link drug response to the genetic information of each patient, known as personalized pharmacogenetics. Therefore, it is relevant to conceive that phenotypic modifications are the consequence of genetic or epigenetic changes that produce simultaneous up‐ and down‐regulation in the expression of different genes, changes that may be induced by the seizures themselves.^6^ Thus, the longer it takes to achieve seizure control, patients who might initially be responders may become drug‐resistant due to genetic changes and changes induced by the cumulative burden of seizure stress.

Seizure control is directly related to a correct choice of ASMs, their dose loading, and frequency of administration, which results in the corresponding balance between doses administered, drug concentrations in blood and the cerebrospinal fluid (CSF), and their therapeutic response, leading to adequate seizure control. Conversely, non‐compliance, insufficient doses, and drug interactions can lead to decreased circulating drug concentrations and, a reduced access to the CNS. Indeed, the amount of drug entering the CNS must be in equilibrium with plasma concentrations, regardless of the route of administration.^7^, ^8^ Naturally, the BBB exerts one of the primary mechanisms controlling such access. On this basis, “peripheral” or pharmacokinetics (PK) conditions (decreased absorption and biodistribution, or increased metabolism or excretion) may lead to a decrease in plasma concentrations of the drug, with inadequate access of ASMs to the CNS. Furthermore, mechanisms acting at the level of the brain parenchyma, including functional and structural modifications of neurons and astrocytes, are considered “central” or pharmacodynamic mechanisms.

ATP‐binding cassette‐transporters (ABC‐t) turn out to be key protagonists in the development of drug resistance, focusing on their functional inhibition to resolve drug resistance. P‐gp represents a clinical target in RE, as previously suggested^9^ and according to its involvement in central and peripheral changes associated with this condition.

## 2 ABC‐TRANSPORTERS AND THE MDR PHENOTYPE

The MDR phenotype not only imposes a clinical problem but also raises the exciting scientific question of how ABC‐t can bind and extrude such a wide range of structurally and functionally unrelated compounds. Several models have been postulated to explain the limited substrate specificity of ABC‐t. These models were initially based on P‐gp observations^5^ but recently extended to account for ABC‐t in organisms such as bacteria, yeast, and eukaryotes.^10^ The fundamental finding was that tumor cells become refractory to multiple chemotherapeutic agents due to the action of P‐gp.^4^ This transporter acts as an energy‐dependent pump that extrudes potentially toxic compounds out of cells and can confer 1000‐fold or greater resistance levels to cells expressing it. ABC‐t constitute a highly conserved superfamily with more than 100 members.^10^ The functional similarity and overlap in substrate “specificity” of the different types of ABC‐t suggest a universal mode of action of these proteins.^11^

**FIGURE 2:**
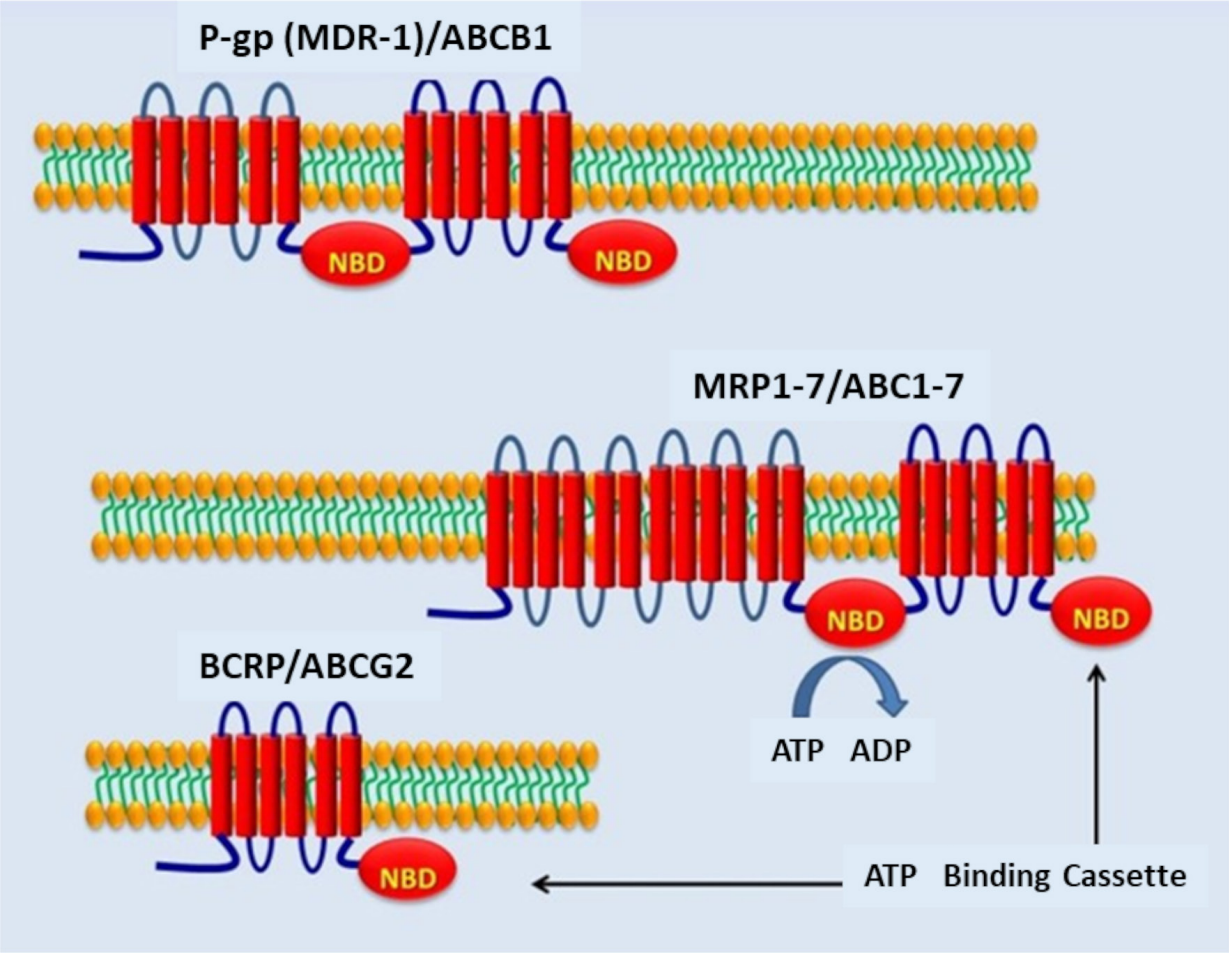
Predicted structure of P‐gp (MDR1)/ABCB1, long‐chain MRP 1‐7 proteins (ABCC1‐7), and BCRP (ABCG2) that need to dimerize to be active. ABC: ATP‐binding cassette; BCRP: breast cancer resistant protein; NBD: nucleotide‐binding domains

Although more than 48 different ABC‐t have been identified in the human genome and divided into seven different classes (A–G) based on structural similarities, only three have been related to the MDR‐phenotype.^12^ In particular, P‐gp, the multidrug‐resistance‐associated proteins (MRP_1–7_), and the BCRP have been associated with the MDR‐phenotype. While P‐gp transports unmodified neutral or positively charged hydrophobic compounds, the substrates of members of the MRP and BCRP transporter subfamily extend to organic anions and Phase II metabolic products^12^ (Figure 2).

## 3 ABC‐T IN THE PERIPHERAL MECHANISM OF DRUG RESISTANCE EPILEPSY

Based on what is known to date, all biological processes related to the drug absorption, biodistribution, metabolism, brain access, and excretion will affect the pharmacokinetic balance between the administered doses of ASMs and their concentration at the site of action. Drug absorption is mainly driven by polarized cell layers, a typical system also observed in excretory organs (liver, kidney, among others) and biological barriers, such as BBB, blood‐testicular, or the feto‐maternal barriers. ABC‐t express constitutively in the gastrointestinal tract, kidney, salivary glands, and VECs of the BBB. In these organs, the ABC‐t plays a central role in drug transport, creating a network of “chemoimmunity” that protects the organism against the accumulation of xenobiotics, including drugs or metabolic compounds.^13^ It is known that their expression depends on interindividual differences (genetic polymorphisms) and the broad spectrum of stimuli (seizures, food, inducing drugs, and the ASMs among others).

**FIGURE 3:**
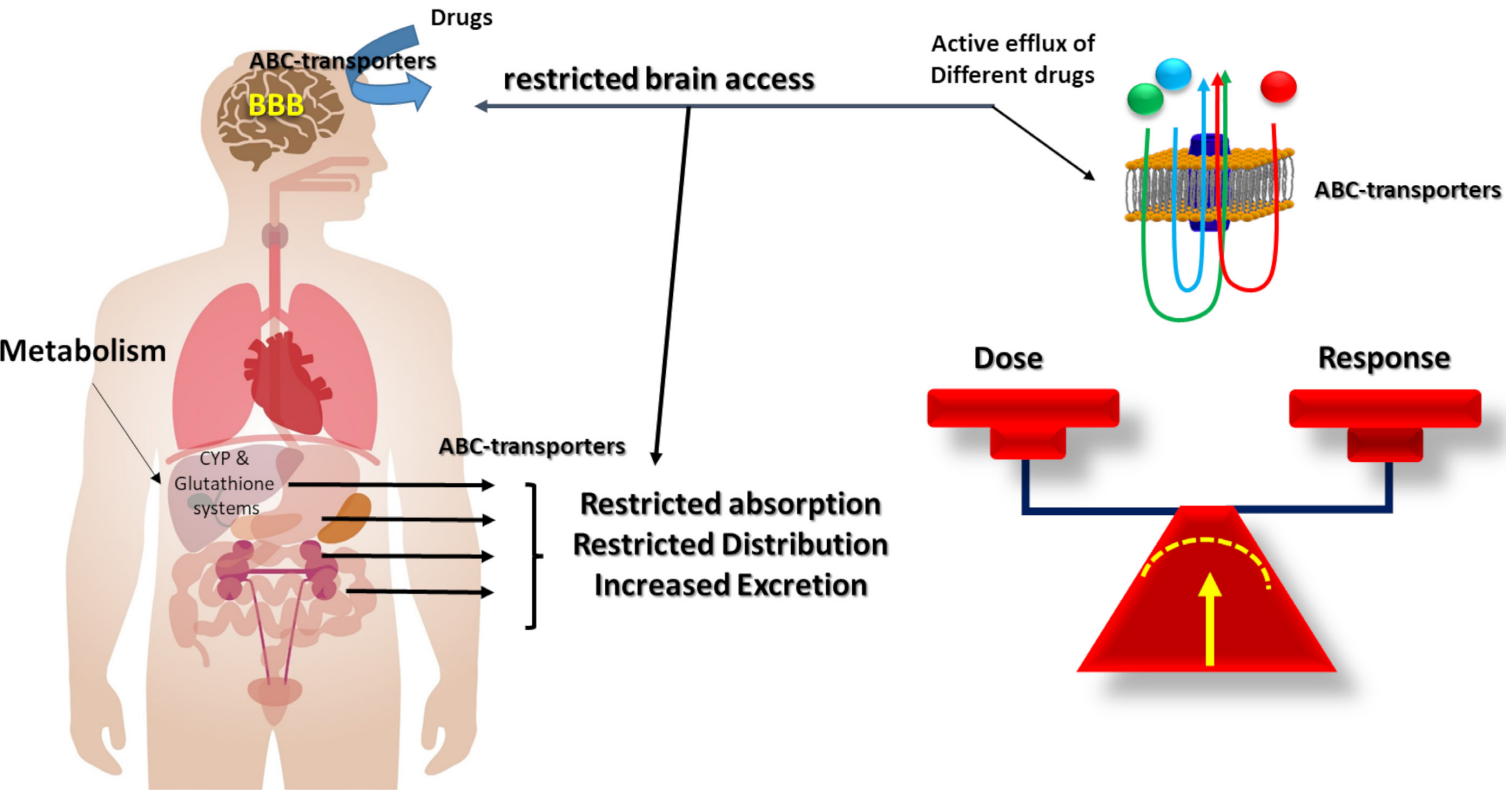
The functional expression of ABC transporters in excretory organs can modify the stability of drug concentrations in plasma and cerebrospinal fluid, a mechanism also related to polymorphisms of these transporters. CYP: cytochrome p450

The small intestine represents the primary site of control of absorption of any ingested compound. Indeed, oral administration is the most popular route for drug administration, as dosing is convenient and noninvasive, and many drugs are well absorbed by the gastrointestinal tract. At this level, ABC‐t located at the apical membrane, including P‐gp, MRP‐2, and BCRP, can drive compounds from inside the cell back into the intestinal lumen, preventing their absorption into the blood. Then, drugs are submitted to metabolism that is classically divided into Phase I (oxidation) and Phase II (conjugation, such as glucuronidation or glutamylation). The synergy between efflux systems and the metabolizing/conjugating enzymes provides a very efficient condition that accounts for rapid drug clearance when these systems are overexpressed. Moreover, both metabolizing and efflux systems share inducers, substrates, and inhibitors. Thus, CYP3A, the main Phase I drug‐metabolizing enzyme, and P‐gp/MDR1 play complementary roles in intestinal drug metabolism^14^ (Figure 3). Although drug metabolism is directly related to the enzymatic activity of the CYP/glutathione system, ABC‐t plays a central role in the amount of drug that is absorbed, biodistributed, and excreted. Together, they control the balance between administered doses and the amount of drug in blood circulation.^14^, ^15^

Hoffmeyer et al demonstrated, for the first time in Caucasian volunteers, a relationship between intestinal MDR‐1 gene expression and the plasma concentration of orally administered digoxin (a drug that undergoes no metabolic changes but is 100% transported by P‐gp). These findings indicated that this feature correlates with a polymorphism in exon 26 (C3435T), where homozygous TT cases had significantly lower duodenal P‐gp expression and higher plasma digoxin levels compared with CC cases. Furthermore, the induction of increased duodenal P‐gp expression after rifampicin (an inducer of P‐gp protein expression) was only observed in CC cases associated with a further decrease in plasma digoxin levels.^16^

**FIGURE 4:**
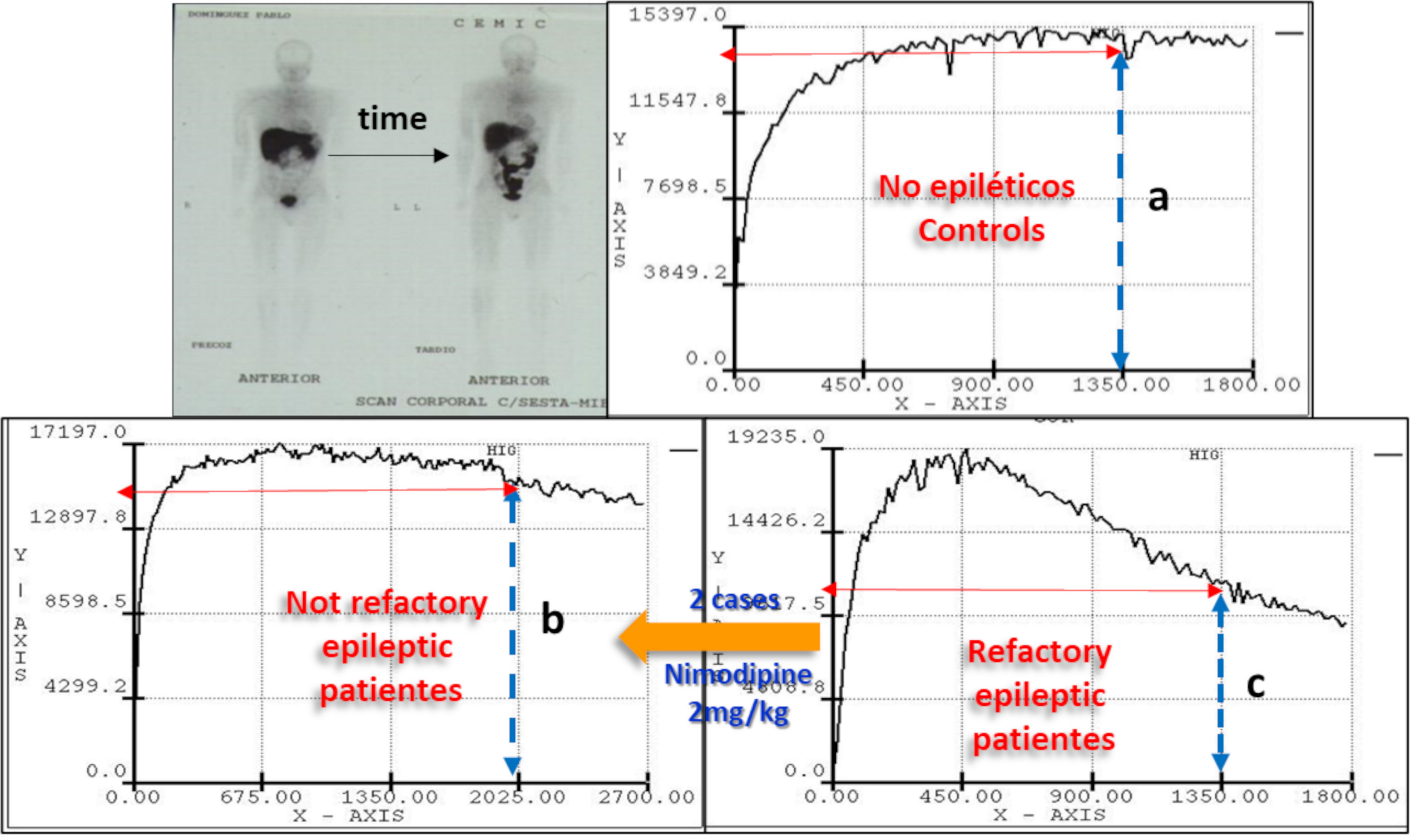
Representation of the hepatic kinetics of ^99m^Tc‐SESTAMIBI in health and epilepsy conditions. Accelerated hepatic washout kinetics of ^99m^Tc‐SESTAMIBI is observed in patients with refractory epilepsy **(c)**, compared to healthy individuals **(a)** and responding (seizure‐free) epileptic patients **(b)**. Two cases with RE, and accelerated kinetics, received nimodipine (2 mg/kg) and recovered similar values to nonrefractory patients. These findings suggest that in cases with uncontrolled repetitive seizures, as in patients with RE, increased functional expression of P‐gp and its possible impact on ASM pharmacokinetics should be suspected

**FIGURE 5:**
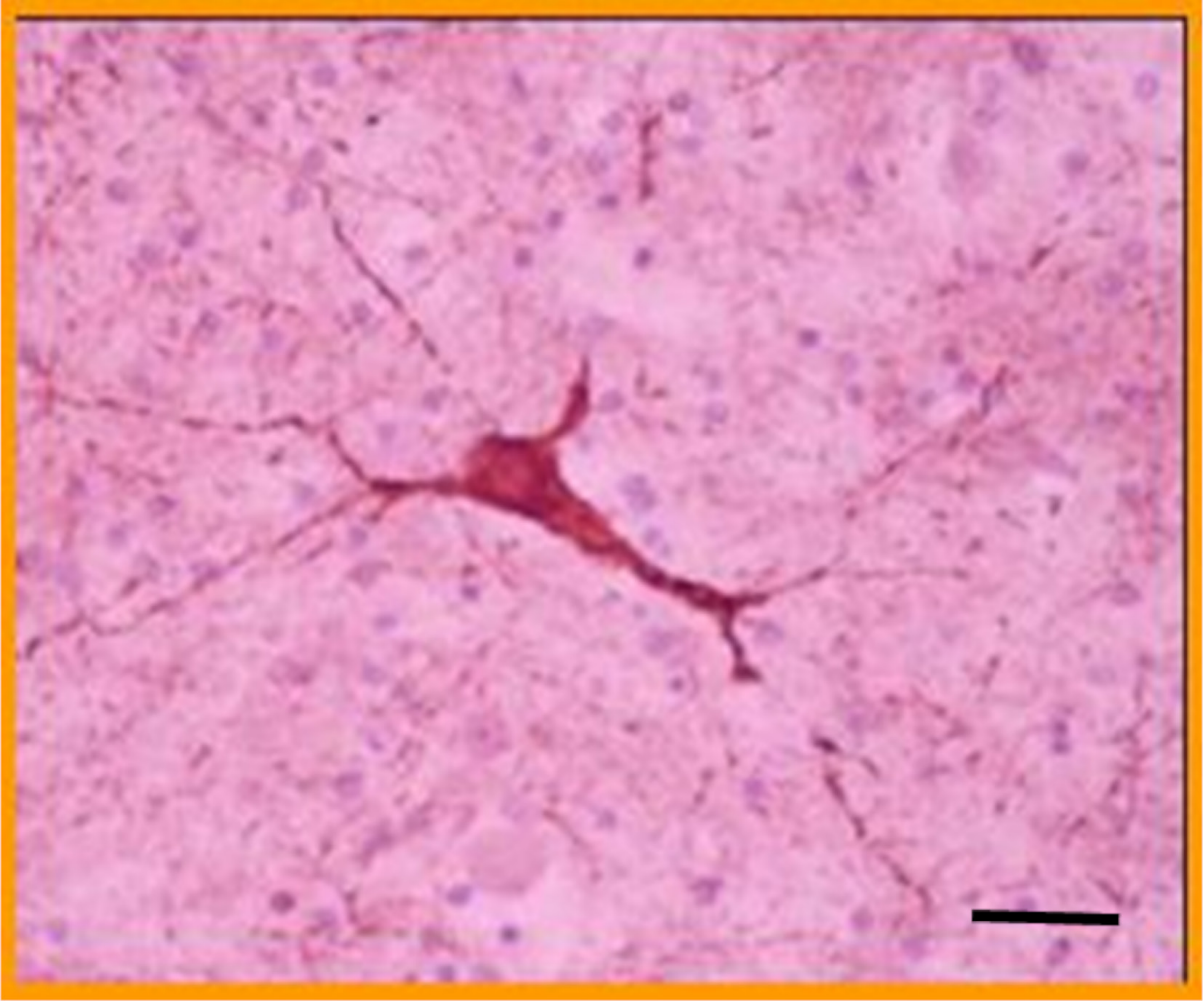
Microphotography of a P‐gp positive dysplastic neuron detected in the epileptogenic area of a case with refractory epilepsy and accelerated hepatic washout of ^99m^Tc‐SESTAMIBI (courtesy of Dr Gustavo Sevlever; Head of the FLENI Pathology Department‐Buenos Aires, Argentina). The same immunostaining result was observed in all 5 surgically treated cases. (P‐gp immunostaining with JSB‐1 monoclonal antibody; bar indicates 200μm; Primary magnification 400X)

In this regard, our group documented that the hepatic clearance of ^99m^Tc‐hexakis‐2‐methoxy‐isobutylisonitrile (^99m‐^Tc‐SESTAMIBI), a P‐gp substrate that behaves similarly to digoxin, was increased in eight patients with RE compared to seven normal subjects and four patients with drug‐responsive epilepsy^17^ (Figure 4). Interestingly, five of these eight cases were treated surgically, and in all of them, P‐gp was expressed in the neurons of the epileptogenic brain area (Figure 5).

Consistent with the previously mentioned report,^16^ it was also shown in Caucasian patients with epilepsy that CC homozygotes of the MDR‐1‐C3435T polymorphism had significantly lower CSF and serum phenobarbital (PHB) concentrations compared with CT heterozygotes and TT homozygotes.^18^ In contrast, in Asian ethnic groups, this genotype‐phenotype correlation was exactly the opposite, showing that Chinese 3435‐TT homozygous patients with epilepsy had decreased plasma carbamazepine (CBZ) levels.^19^, ^20^ This observation was also reported in a systematic review and meta‐analysis.^21^

These data indicate the usefulness of the pharmacogenetic evaluation of these polymorphisms for appropriate therapeutic prescription according to the corresponding ethnic group. Additionally, a valuable tool to identify this particular pharmacological behavior is therapeutic ASMs monitoring showing their persistent decreases in plasma levels,^22^, ^23^ or inversely, detecting their higher salivary excretion,^24^ in patients with RE.

In a clinical study of 70 patients treated with oral phenytoin (PHT), the mean plasma concentration of free drug was significantly higher in responsive than patients with a partial response.^25^ These and other similar results allowed Tang et al to postulate the “pharmacokinetic hypothesis” and suggest that increased peripheral expression of ABC‐t and metabolizing enzymes could play a synergistic effect, lowering plasmatic levels of ASMs.^2^

Our group has also documented a high frequency of persistent low levels (PLLs) of the most common ASMs, such as PHT, PHB, valproic acid (VA), and CBZ, in a population of pediatric epileptic patients (PEPs). In this study, we retrospectively analyzed 21,040 plasma levels of the ASMs mentioned above from 3,279 PEPs. In this population, PLLs of PHT were detected in inpatients (71.7%) and outpatients (74.1%), whereas PLLs of PHB, VA, and CBZ were also detected in a smaller proportion. In some patients, PLL of at least one ASM during long periods of hospitalization was documented as a dominant feature for four drugs evaluated.^26^ This particular observation indicates that PLL is not only a compliance issue but should be considered as a pharmacokinetic change related to clinical conditions. Unfortunately, some clinical epileptologists do not interpret these data as altered pharmacokinetic behavior but as a laboratory error and therefore decide to withdraw the drug found with PLL in plasma.

Apart from the mechanisms that regulate drug absorption, ABC‐t hepatic and renal expression plays a central role in regulating drug plasma levels, metabolism and excretion. In addition, ASMs can modify the ABC‐t expression. It is known that chronic treatment with ASMs such as PHT can induce a transient overexpression of P‐gp in the rat liver, which can be reversed if the ASM is removed from the body.^27^ Similarly, different stimuli, such as hormones, oncogenes, and transcription factors, known to be involved in apoptosis, stress, inflammation, and hypoxia (eg, p53, NFkB, NF‐IL6, AP‐1, HIF‐1α), can induce overexpression of ABC‐t at the level of excretory organs. Interestingly, some of these factors can also induce a simultaneous de novo expression of ABC‐t in previously nonexpressive cells, such as neurons and cardiomyocytes.^28^ Previous experimental studies have shown that seizures induce P‐gp expression not only at the brain level (neurons, astrocytes, VECs)^29^ but also in peripheral organs directly related to drug clearance, such as the liver and kidney,^30^ or related to sudden unexpected death in epilepsy (SUDEP), such as the heart.^28^, ^31^

According to the previous information, the change in the functional expression profile of ABC‐t in peripheral organs may produce a new scenario and generate pharmacoresistance by reducing the available ASM concentrations.

## 4 ABC‐T IN THE CENTRAL MECHANISM OF DRUG‐RESISTANCE EPILEPSY: IS P‐GP EXPRESSION IN NEURONAL MEMBRANES AN EPILEPTOGENIC FACTOR?

**FIGURE 6:**
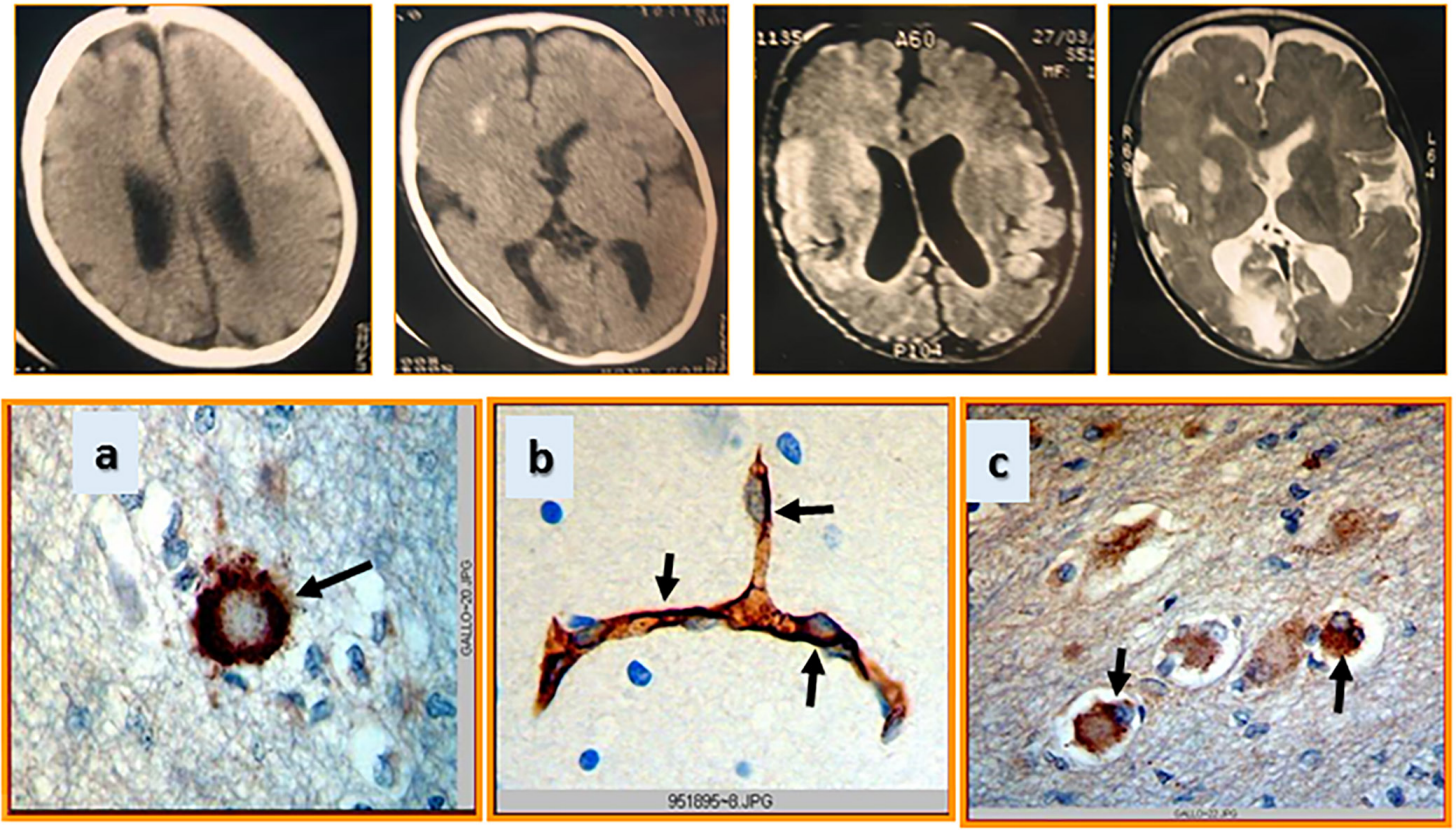
Upper panel: computed tomography and magnetic resonance images of the brain showing a cortical dysplasia lesion. Lower panel: a) immunostaining showing the major vault protein detected in a ballooned neuron; b) high expression of breast cancer–resistant protein is detected in the blood–brain barrier, and in c) several ballooned neurons from the epileptogenic brain area of a child with refractory epilepsy due to cortical dysplasia

P‐glycoprotein is normally expressed in brain VECs.^32^, ^33^ However, this expression could be downregulated under different conditions (eg, in Parkinson's disease and in the advanced stages of neurodegenerative processes such as Alzheimer's disease).^34^, ^35^ In contrast, other studies have indicated that high levels of P‐gp transcripts or elevated P‐gp immunoreactivity have been detected in endothelial and glial cells of brain samples surgically removed from patients with RE.^36^, ^37^, ^38^ The first description of P‐gp expression in abnormal neurons from epileptogenic brain areas was reported in dysplastic neurons from a child with RE due to tuberous sclerosis.^23^ This particular neuronal expression was subsequently described in another report of a pediatric case of drug resistance.^22^ Interestingly, both cases shared the PLL of ASMs. Additionally, other reports have demonstrated that P‐gp, MRP, BCRP, and a NOT‐ABCt, such as the major vault protein, were overexpressed in brain samples from some other PEPs with RE^39^, ^40^, ^41^, ^42^ (Figure 6) and adult patients with temporal lobe epilepsy.^43^, ^44^, ^45^

Similar results were also described in a pediatric case with focal cortical dysplasia,^46^ in another case with transmantle cortical dysplasia,^47^ and some patients with epilepsy due to brain tumors.^48^, ^49^ Consistent with these clinical findings, neuronal expression of P‐gp was also observed in different experimental models of seizures^29^, ^50^ and brain hypoxia,^51^ where administration of nimodipine restored PHT PK in the hippocampus, with complete recovery of protection against induced seizures.^52^

**FIGURE 7:**
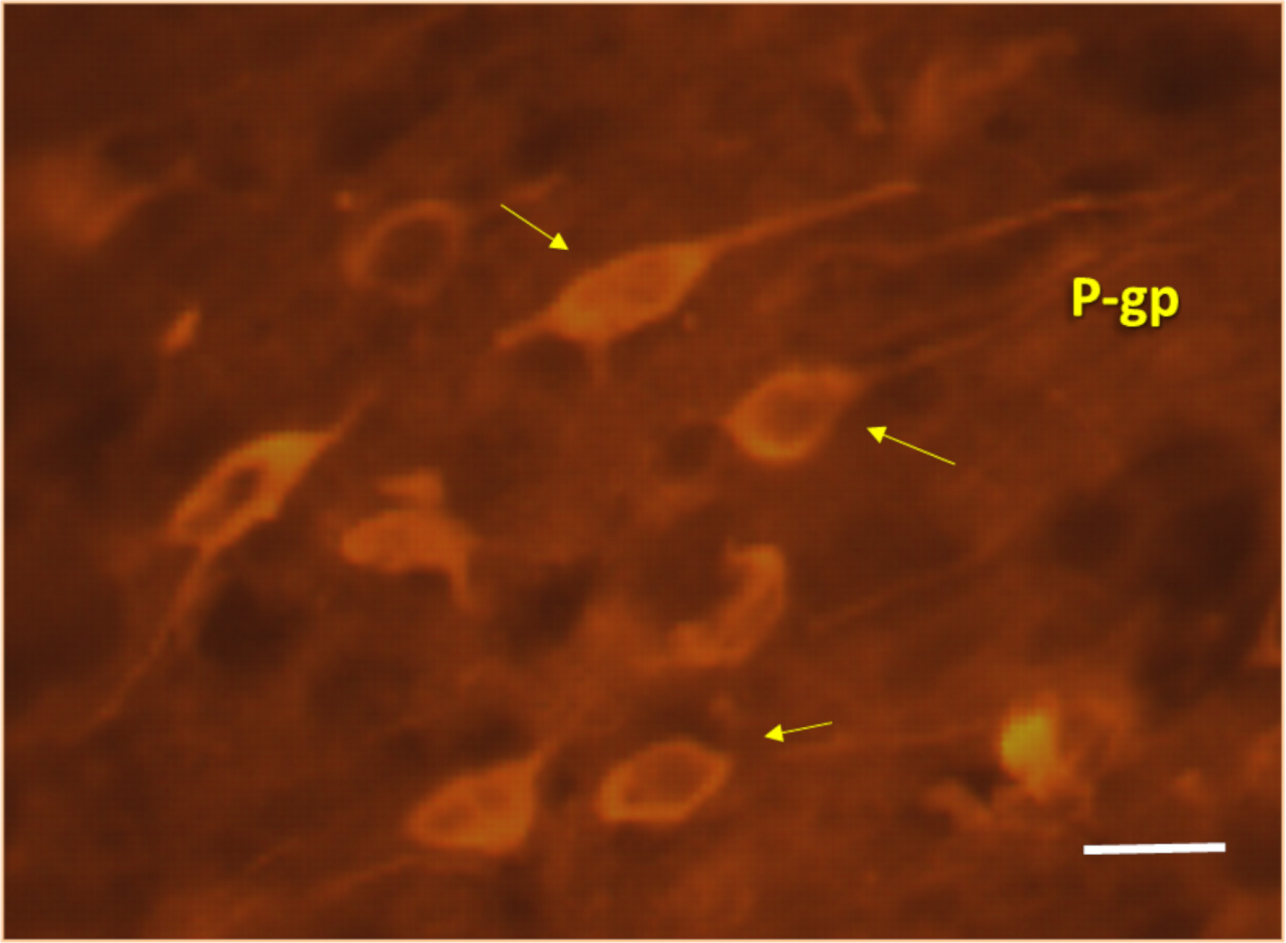
Microphotography showing high detection in neurons of P‐gp immunostaining with monoclonal antibody (clone C494‐Signet Laboratories, Dedham, MA). Notice the neuronal expression of P‐gp after repetitive induced experimental seizures. Similar images can also be observed in hypoxia‐induced brain samples (bar indicates 200μm; Primary magnification 400X)

Because most ASMs act by binding to receptors present on the outer side of neuronal membranes, the presence of P‐gp on these membranes should not alter the binding of ASMs to their therapeutic targets. Therefore, we should ask what role P‐gp plays in these membranes. Regardless of the active efflux role of P‐gp—exporting drugs outside of cells and organs—its expression in neuronal membranes is not consistent with this transport function, so another role should be postulated. This intriguing question arose when our experimental findings showed that 100% of animals were resistant to PHT only after 7 days of 3‐mercapto‐propionic acid–induced seizures, which was associated with P‐gp predominantly expressed in the neurons^29^ (Figure 7).

**FIGURE 8:**
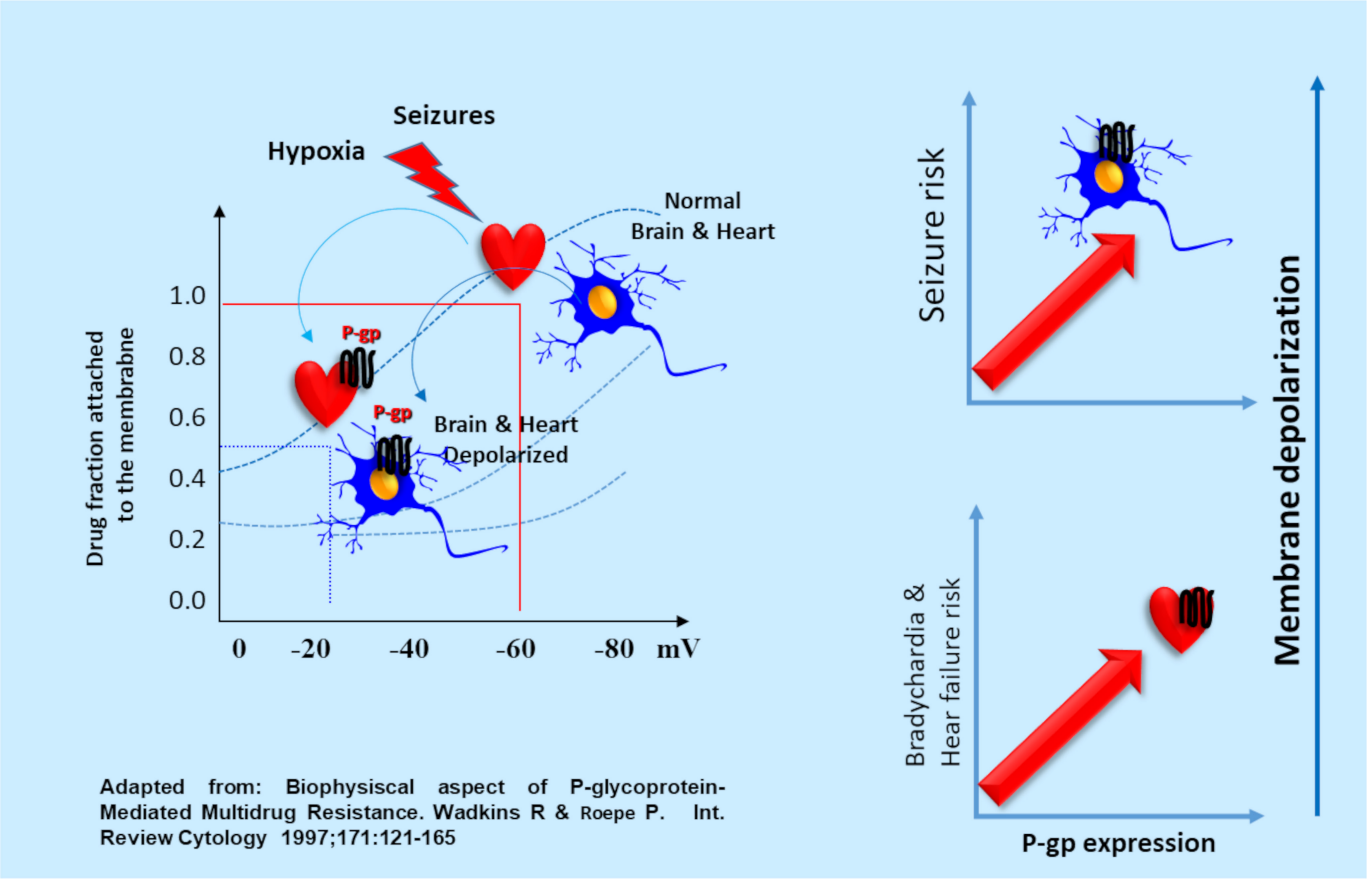
Diagram showing the hypothetical role of P‐gp in the resting membrane potential under normal and epileptic conditions. Similar to that demonstrated in tumor cells, we suggest that normal neurons and cardiomyocytes have a resting membrane potential of −60 mV, but seizures/hypoxia damage will induce P‐gp expression in both cell types. The P‐gp expression will lead to a depolarized membrane potential of −20 mV. Therefore, the higher the P‐gp expression in the membrane of neurons and cardiomyocytes, the higher will be the risk of status epilepticus and heart failure, resulting in sudden unexpected death in epilepsy (SUDEP)

According to these results, we suggest that P‐gp in neurons might play a role in the development of more severe seizures. In this regard, an alternative mechanism to the classical pumping function of P‐gp was observed in tumor cells expressing the MDR‐1 gene. These cells have a significantly low membrane potential (ΔΨ0 = −10 to −20) compared with the physiological potential (ΔΨ0 = −60 mV)^53^, ^54^ and facilitating neuronal depolarization (Figure 8). Consistent with this hypothesis, our group demonstrated that overexpression of brain P‐gp contributes to progressive depolarization seizure‐related membranes in the hippocampus and neocortex in a rat model of pentylenetetrazole (PTZ)‐induced repetitive seizures, and only the combined administration of PHT and nimodipine (a P‐gp antagonist) restored normal membrane potential.^55^ However, the intimate mechanism of this depolarizing property of P‐gp, observed in both tumor cells and brain tissue sections, has not yet been elucidated.

Seizures could induce P‐gp expression through different mechanisms. In the early 1970s, cerebral hypoxia was described as causing seizures,^56^ a condition that induces mechanisms of either rescue and survival of eukaryotic cells or death by apoptotic pathway.

This dual response to hypoxia (acute or chronic) is mediated by the transcription factor HIF1α (hypoxia‐inducible factor 1‐α), which was initially described by Greg L. Semenza, who won the 2019 Nobel Prize for this discovery. HIF‐1α plays a master role in the stimulation or repression of an extensive list of genes that modify the functionality of cells under conditions of hypoxia.^57^ Among these genes, erythropoietin (EPO) and erythropoietin receptor (EPO‐R) genes are also upregulated by HIF‐1α.^58^ P‐gp can be induced not only by seizures through glutamate and cyclooxygenase‐2 signaling^59^ but also by HIF‐1α,^60^ and hypoxic neurons will also express both P‐gp and EPO‐R.^58^, ^61^

However, we have been able to show the overexpression of P‐gp and EPO‐R in neurons after convulsive stress, accompanied by a translocation of HIF‐1α at the nuclear level, and EPO administration protected against ischemic brain damage and inhibited P‐gp‐mediated Rho‐123 transport.^62^ This information leads to support EPO administration as a novel strategy to control disorders associated with the overexpression of P‐gp such as hypoxia and RE.^63^

## 5 BRAIN INFLAMMATION, ABC TRANSPORTERS, AND BLOOD‐BRAIN BARRIER DYSFUNCTION

Sequential mechanisms including excitotoxicity, depolarization, inflammation, and apoptotic death can be observed after different brain insults, such as trauma, hypoxia, seizures, among others.^64^ In all cases, immunological and inflammatory mechanisms may also be involved in the pathogenesis of epilepsy, where critical mediators of this process include interleukin IL‐1β, IL‐6, tumor necrosis factor‐α, as well as oxidative stress.^65^, ^66^, ^67^ Interestingly, induced overexpression of P‐gp at the BBB and neuronal levels has been documented in these scenario.^61^ In addition, alterations of the brain functional expression of some other transporters have been reported, such as the glucose transporter GLUT1, with decreased brain glucose uptake and hypometabolism in the epileptic area.^68^ Similarly, altered expression of aquaporin‐4 (AQP4) has also been demonstrated in both human temporal lobe epilepsy and experimental seizure models. Typically, AQP4 is expressed at the astrocyte end feet in the BBB. In addition, parenchymal expression of AQP4 is increased in sclerotic hippocampi but reduced in the BBB. Lack of AQP4 in the BBB contributes to water efflux and impaired K^+^ buffering. Interestingly, impaired K^+^ buffering affects brain energetics and homeostasis via an ATP‐dependent mechanism. In rodent and human epileptic brains, K^+^ buffering is compromised due to reduced expression of Kir 4.1.^67^ In a recent review, the relationship between neuroinflammation, BBB dysfunction, brain overexpression of ABC‐t, and RE was updated.^69^

The first evidence showing transient expression of P‐gp in astrocytes was reported after intracerebroventricular injection of kainate, and immunostaining was detected as early as 1 day postinjection, with a peak at 2 weeks, and still visible up to 10 weeks postinjection.^70^ Moreover, the first transient neuronal P‐gp expression was reported in a rat model of ischemia by partial cortical devascularization. This transitory expression showed a changing pattern in the course of time with a maximum at 7 days after injury and subsequently reduced to undetectable levels at 28 days after injury.^71^ These two pioneering experiments strongly suggested that inducible P‐gp expression can be reversed as long as normality is restored and the initial short‐term insult is not repeated. Consequently, repetitive inducing insults are expected to generate a long‐lasting increasing level of P‐gp expression,^28^, ^29^, ^55^ as a window of therapeutic resistance.

## 6 EXPRESSION OF P‐GLYCOPROTEIN IN THE HEART AND ITS POTENTIAL ROLE IN SUDEP DEVELOPMENT

Sudden unexpected death in epilepsy, by definition, is a sudden and unexpected death in a person with epilepsy for which no obvious cause has been found to date^72^ and is one of the most feared and catastrophic complications of epilepsy. It is urgent to identify the biological mechanisms that can trigger this condition and therefore define the predictive biomarkers of SUDEP.

Evidence suggests that SUDEP may be a consequence of heart failure due to elevated hypoxic stress secondary to ictal hypoxemia (IH), and excessive sympathetic overstimulation triggering neurocardiogenic injury. The severity of IH appeared independent from the age group and from seizure type and is probably the major clinical concern for its correlation with potentially life‐threatening cardiorespiratory alterations and SUDEP.^73^

Several risk factors have been associated with SUDEP, most notably the severity and frequency of generalized tonic‐clonic seizures (GTCS). In this regard, the concept of “epileptic heart” has recently been reported as “…a heart and coronary vasculature damaged by chronic epilepsy due to repeated hypoxemia and increased catecholamines leading to electrical and mechanical heart dysfunction.”^74^ These repetitive injuries would be affecting the electrical properties of the myocardium, causing heart failure with fatal arrhythmia (bradycardia).

It is important to emphasize that under normal conditions, P‐gp is low expressed in the endothelial cells of capillaries and arterioles in the healthy heart, but absent in cardiomyocytes.^75^, ^76^ On the other hand, it has been assumed that seizures can act as hypoxic stress and induce overexpression of P‐gp and EPO‐R in a HIF‐1α dependent manner, not only in the brain (neurons, astrocytes, VECs of BBB)^61^ but also in cardiomyocytes.^77^, ^78^ Experimental evidence suggests that the highly cumulative burden of convulsive stress (high frequency of GTCS or status epilepticus) results in a hypoxic cardiac insult, where P‐gp expression^79^, ^80^ could play a depolarizing role in cardiomyocyte membranes.^31^

In addition, the Kir channels are molecular regulators of the membrane potential in cardiomyocytes to control heart rate, and whose genetic variants have been related to epilepsy^81^ and cardiac dysfunction.^82^ Kir controls cellular excitability in the heart by acting toward the repolarization phase of the cardiac action potential. Notably, a significant decrease in cardiac Kir channel mRNA and protein expression was observed in PTZ‐kindled rats.^83^ By using the same PTZ‐epileptic model, seizures increased P‐gp and reduced Kir expression, producing a collaborative action to stabilize cardiomyocyte membrane depolarization.^6^ Accordingly, we suggest that repetitive convulsive events (assumed as repetitive ischemic events) could generate long‐lasting P‐gp expression at the cardiac level as a window of susceptibility to functional heart failure.

Based on the above, we have reported that repetitive status epilepticus induces a hypoxic condition in the heart with low retention of ^99m^‐Tc‐MIBI associated with severe bradycardia and a high rate of spontaneous death.^79^ Pioneering experimental evidence described that cardiac hypoxia was directly related to high P‐gp expression in cardiomyocytes in the ischemic area, associated with low retention of ^99m^‐Tc‐MIBI.^78^, ^79^ Recently, our group suggested that the intimate pathophysiological relationship between seizures and hypoxia affects the brain and the heart, elevating the risk of SUDEP.^84^ We hypothesize that an acute fatal heart rhythm alteration could result from severe hypoxic stress produced by repetitive seizures, where P‐gp plays a depolarizing role in cardiomyocytes.

Therefore, we suggest a vicious circle: cardiac disorders may induce BBB alterations, status epilepticus, seizures, and epilepsy. In contrast, seizures, status epilepticus, and uncontrolled epilepsy may induce cardiac dysfunctions, recalling the “chicken and egg” puzzle concept coined by Friedman,^85^ with a high risk of death (SUDEP). The potential use of ^99m^Tc‐SESTAMIBI cardio‐SPECT, both in resting and effort conditions, could represent noninvasive technologies to detect the risk of acute cardiological failure, and thus, be the telltale heart (Edgar Allan Poe) of SUDEP, in patients with RE.^86^

Finally, it is essential to highlight recent advances in using cannabinoids for the treatment of RE.^87^ Several mechanisms have been proposed by which cannabinoids could exert their beneficial effects for the most severe types of epilepsy.^88^, ^89^ By docking studies, our group recently demonstrated that cannabidiol has binding sites on P‐gp and induces an inhibitory effect on the efflux transport of Rho‐123 (a P‐gp substrate) in cultured cells,^90^ such as that induce by nimodipine.^52^ This finding gives molecular consistency to the efficacy of cannabidiol (as adjuvant therapy) in the control of most severe refractory epilepsies.^91^

## 7 CONCLUSIONS AND REMARKS

Based on what has been described, central ABC‐t expression in neurons could play a role at central level in the epileptogenesis. Additionally, peripheral induction of ABC‐t in the small intestine may decrease drugs absorption, whereas in the liver, kidney, and BBB may increase drug clearance, all affecting their PK, decreasing plasma drug levels, and reducing access of ASMs to the brain.^92^, ^93^ In addition, induction of P‐gp expression in the heart may have a potential role in heart failure and SUDEP. Both central and peripheral mechanisms may occur independently but may be also concomitants according with the severity and frequency of seizures. Therefore, we suggested that P‐gp and the other ABC‐t related to MDR phenotype should be new targets for the treatment of RE.^9^

In summary, we propose that the transporter hypothesis for drug‐resistance epilepsy should consider the following conditions:

- At the peripheral level, the increased expression of ABC‐t induces PK alterations by changes ASM absorption, biodistribution, brain access, and excretion.
- At the central level, ABC‐t (particularly P‐gp) could play a pivotal role by modifying neuronal functional properties associated with membrane depolarization. This situation results in pharmacodynamic alterations related not only to the drug‐resistance phenotype but also to epileptogenesis, heart failure and SUDEP, which represents a peripheral fatal phenomenon.

## CONFLICT OF INTEREST

Neither of the authors has any conflict of interest to disclose. We confirm that we have read the Journal's position on issues involved in ethical publication and affirm that this report is consistent with those guidelines.

## ETHICAL APPROVAL

We confirm that we have read the journal's position on issues involved in ethical publication and affirm that this report is consistent with those guidelines.

## References

[1] Sisodiya SM . Genetics of drug resistance. Epilepsia. 2005;46(Suppl 10):33–8. 10.1111/j.1528-1167.2005.00356.x 16359469

[2] Tang F , Hartz AMS , Bauer B . Drug‐resistant epilepsy: multiple hypotheses, few answers. Front Neurol. 2017;8:301. 28729850 10.3389/fneur.2017.00301 PMC5498483

[3] Pérez‐Pérez D , Frías‐Soria CL , Rocha L . Drug‐resistant epilepsy: From multiple hypotheses to an integral explanation using preclinical resources. Epilepsy Behav. 2021;121(Pt B):106430. 31378558 10.1016/j.yebeh.2019.07.031

[4] Ling V . Does P‐glycoprotein predict response to chemotherapy? J Natl Cancer Inst. 1989;81(2):84–5. 2562857 10.1093/jnci/81.2.84

[5] Juliano RL , Ling V . A surface glycoprotein modulating drug permeability in Chinese hamster ovary cell mutants. Biochim Biophys Acta. 1976;455:152–62. 990323 10.1016/0005-2736(76)90160-7

[6] Auzmendi J , Akyuz E , Lazarowski A . The role of P‐glycoprotein (P‐gp) and inwardly rectifying potassium (Kir) channels in sudden unexpected death in epilepsy (SUDEP). Epilepsy Behav. 2021;121(Pt B):106590. 31706919 10.1016/j.yebeh.2019.106590

[7] Rabinowicz AL , Salvat JM , Leiguarda RC , Demonty F , Salvat F , Cervio A , et al. Use of antiepileptic drugs in nontraumatic neurosurgical procedures. Is there any best route and time of administration? Clin Neuropharmacol. 1997;20(5):438–41. 9331520 10.1097/00002826-199710000-00008

[8] König J , Müller F , Fromm MF . Transporters and drug‐drug interactions: important determinants of drug disposition and effects. Pharmacol Rev. 2013;65(3):944–66. 23686349 10.1124/pr.113.007518

[9] Robey WR , Lazarowski A , Bates SE . P‐glycoprotein—a clinical target in drug‐refractory epilepsy? Mol Pharmacol. 2008;73(5):1343–6. 18314494 10.1124/mol.108.046680

[10] Srikant S . Evolutionary history of ATP‐binding cassette proteins. FEBS Lett. 2020;594(23):3882–97. 33145769 10.1002/1873-3468.13985

[11] Gottesman MM , Pastan I . Biochemistry of multidrug resistance mediated by the multidrug transporter. Ann Rev Biochem. 1993;62:385–427. 8102521 10.1146/annurev.bi.62.070193.002125

[12] Dean M , Rzhetsky A , Allikmets R . The human ATP‐binding cassette (ABC) transporter superfamily. Genome Res. 2001;11:1156–66. 11435397 10.1101/gr.184901

[13] Sarkadi B , Homolya L , Szakács G , Váradi A . Human multidrug resistance ABCB and ABCG transporters: participation in a chemoimmunity defense system. Physiol Rev. 2006;86:1179–236. 17015488 10.1152/physrev.00037.2005

[14] Szakács G , Váradi A , Ozvegy‐Laczka C , Sarkadi B . The role of ABC transporters in drug absorption, distribution, metabolism, excretion and toxicity (ADME–Tox). Drug Discov Today. 2008;13(9–10):379–93. 18468555 10.1016/j.drudis.2007.12.010

[15] Koehn LM , Dziegielewska KM , Habgood MD , Huang Y , Saunders NR . Transfer of rhodamine‐123 into the brain and cerebrospinal fluid of fetal, neonatal, and adult rats. Fluids Barriers CNS. 2021;18(1):6. 33557872 10.1186/s12987-021-00241-8 PMC7871379

[16] Hoffmeyer S , Burk O , von Richter O , Arnold HP , Brockmöller J , Johne A , et al. Functional polymorphisms of the human multidrugresistance gene: multiple sequence variations and correlation of one allele with P‐glycoprotein expression and activity in vivo. Proc Natl Acad Sci USA. 2000;97:3473–8. 10716719 10.1073/pnas.050585397 PMC16264

[17] Vazquez SE , D’Giano C , Carpintiero S , Coronel K , Ugarnes G , Lazarowski A . Increase 99mTc‐SESTAMIBI (MIBI) liver clearance could identified epileptic pharmacoresistant patients. A preliminary study. Annual Meeting of the American Epilepsy Society. Epilepsia. 2004;45:120.

[18] Basic S , Hajnsek S , Bozina N , Filipcic I , Sporis D , Mislov D , et al. The influence of C3435T polymorphism of ABCB1 gene on penetration of phenobarbital across the blood‐brain barrier in patients with generalized epilepsy. Seizure. 2008;17:524–30. 18329296 10.1016/j.seizure.2008.01.003

[19] Seo T , Ishitsu T , Ueda N , Nakada N , Yurube K , Ueda K , et al. ABCB1 polymorphisms influence the response to antiepileptic drugs in Japanese epilepsy patients. Pharmacogenomics. 2006;7(4):551–61. 16753003 10.2217/14622416.7.4.551

[20] Meng H , Guo G , Ren J , Zhou H , Ge Y , Guo Y . Effects of ABCB1 polymorphisms on plasma carbamazepine concentrations and pharmacoresistance in Chinese patients with epilepsy. Epilepsy Behav. 2011;21(1):27–30. 21493161 10.1016/j.yebeh.2011.02.015

[21] Chouchi M , Kaabachi W , Klaa H , Tizaoui K , Ben‐Youssef Turki I , Hila L . Relationship between ABCB1 3435TT genotype and antiepileptic drugs resistance in Epilepsy: updated systematic review and meta‐analysis. BMC Neurol. 2017;17:32. 28202008 10.1186/s12883-017-0801-x PMC5311838

[22] Lazarowski A , Massaro M , Schteinschnaider A , Intruvini S , Sevlever G , Rabinowicz A . Neuronal MDR‐1 gene expression and persistent low levels of anticonvulsants in a child with refractory epilepsy. Ther Drug Monit. 2004;26:44–6. 14749549 10.1097/00007691-200402000-00010

[23] Lazarowski A , Sevlever G , Taratuto A , Massaro M , Rabinowicz A . Tuberous sclerosis associated with MDR1 gene expression and drug‐resistant epilepsy. Pediatr Neurol. 1999;21:731–4. 10580886 10.1016/s0887-8994(99)00074-0

[24] Fagiolino P , Vázquez M , Maldonado C , Ruiz ME , Volonté MG , Orozco‐Suárez S , et al. Usefulness of salivary drug monitoring for detecting efflux transporter overexpression. Curr Pharm Des. 2013;19(38):6701. 23530513 10.2174/13816128113199990368

[25] Iwamoto T , Kagawa Y , Naito Y , Kuzuhara S , Okuda M . Clinical evaluation of plasma free phenytoin measurement and factors influencing its protein binding. Biopharm Drug Dispos. 2006;27:77–84. 16308884 10.1002/bdd.486

[26] Czornyj L , Cáceres Guido P , Bramuglia G , Rodiño A , Feria‐Romero I , Lazarowski A . High incidence of persistent subtherapeutic levels of the most common ASMs in children with epilepsy receiving polytherapy. Epilepsy Res. 2018;148:107–14. 10.1016/j.eplepsyres.2018.09.008 30279018

[27] Alvariza S , Fagiolino P , Vázquez M , Feria‐Romero I , Orozco‐Suárez S . Chronic administration of phenytoin induces efflux transporter overexpression in rats. Pharmacol Rep. 2014;66(6):946–51. 25443719 10.1016/j.pharep.2014.06.007

[28] Auzmendi J , Merelli A , Girardi E , Orozco‐Suarez S , Rocha L , Lazarowski A . Progressive heart P‐glycoprotein (P‐gp) overexpression after experimental repetitive seizures (ERS) associated with fatal status epilepticus (FSE). Is it related with SUDEP? Mol Cell Epilepsy. 2014;1:43–51. https://www.smartscitech.com/index.php/MCE/article/view/935

[29] Lazarowski A , Ramos AJ , García‐Rivello H , Brusco A , Girardi E . Neuronal and glial expression of the multidrug resistance gene product in an experimental epilepsy model. Cell Mol Neurobiol. 2004;24(1):77–85. 15049512 10.1023/B:CEMN.0000012726.43842.d2 PMC11529958

[30] Guo Y , Jiang L . Drug transporters are altered in brain, liver and kidney of rats with chronic epilepsy induced by lithium–pilocarpine. Neurol Res. 2010;32:106–12. 19570321 10.1179/174313209X408954

[31] Auzmendi J , Buchholz B , Salguero J , Cañellas C , Kelly J , Men P , et al. Pilocarpine‐induced status epilepticus is associated with P‐glycoprotein induction in cardiomyocytes, electrocardiographic changes, and sudden death. Pharmaceuticals (Basel). 2018;11(1):21. 10.3390/ph11010021 PMC5874717 29462915

[32] Verscheijden LFM , van Hattem AC , Pertijs JCLM , de Jongh CA , Verdijk RM , Smeets B , et al, Wildt SN . Developmental patterns in human blood‐brain barrier and blood‐cerebrospinal fluid barrier ABC drug transporter expression. Histochem Cell Biol. 2020;154(3):265–73. 10.1007/s00418-020-01884-8 32448916 PMC7502061

[33] Enrique AV , Di Ianni ME , Goicoechea S , Lazarowski A , et al. New anticonvulsant candidates prevent P‐glycoprotein (P‐gp) overexpression in a pharmacoresistant seizure model in mice. Epilepsy Behav. 2021;121(Pt B):106451. 31420290 10.1016/j.yebeh.2019.106451

[34] Bartels AL , Willemsen ATM , Kortekaas R , de Jong BM , de Vries R , de Klerk O , et al. Decreased blood‐brain barrier P‐glycoprotein function in the progression of Parkinson’s disease, PSP and MSA. J Neural Transm (Viena). 2008;115:1001–9. 10.1007/s00702-008-0030-y PMC2468317 18265929

[35] Vogelgesang S , Cascorbi I , Schroeder E , Pahnke J , Kroemer HK , Siegmund W , et al. Deposition of Alzheimer’s beta‐amyloid is inversely correlated with P‐glycoprotein expression in the brains of elderly non‐demented humans. Pharmacogenetics. 2002;12:535–41. 12360104 10.1097/00008571-200210000-00005

[36] Sisodiya SM , Lin WR , Squier MV , Thom M . Multidrug‐resistance protein 1 in focal cortical dysplasia. Lancet. 2001;357:42–3. 11197364 10.1016/s0140-6736(00)03573-x

[37] Sisodiya SM , Lin WR , Harding BN , Squier MV , Thom M . Drug resistance in epilepsy: expression of drug resistance proteins in common causes of refractory epilepsy. Brain. 2002;125(Pt 1):22–31. 11834590 10.1093/brain/awf002

[38] Tishler DM , Weinberg KI , Hinton DR , Barbaro N , Annett GM , Raffel C . MDR1 gene expression in brain of patient with medically intractable epilepsy. Epilepsia. 1995;36:1–6. 10.1111/j.1528-1157.1995.tb01657.x 8001500

[39] Lazarowski A , Lubieniecki F , Camarero S , Pomata H , Bartuluchi M , Sevlever G , et al. Multidrug resistance proteins in tuberous sclerosis and refractory epilepsy. Pediatr Neurol. 2004;30:102–6. 14984901 10.1016/S0887-8994(03)00407-7

[40] Lazarowski AJ , Lubieniecki FJ , Camarero SA , Pomata HH , Bartuluchi MA , Sevlever G , et al. New proteins configure a brain drug resistance map in tuberous sclerosis. Pediatr Neurol. 2006;34:20–4. 16376273 10.1016/j.pediatrneurol.2005.06.008

[41] Brukner AM , Billington S , Benifla M , Nguyen TB , Han H , Bennett O , et al. Abundance of P‐glycoprotein and breast cancer resistance protein measured by targeted proteomics in human epileptogenic brain tissue. Mol Pharm. 2021;18(6):2263–73. 10.1021/acs.molpharmaceut.1c00083 34008992 PMC8488956

[42] Banerjee Dixit A , Sharma D , Srivastava A , Banerjee J , Tripathi M , Prakash D , et al. Upregulation of breast cancer resistance protein and major vault protein in drug‐resistant epilepsy. Seizure. 2017;47:9–12. 28273590 10.1016/j.seizure.2017.02.014

[43] Ak H , Ay B , Tanriverdi T , Sanus GZ , Is M , Sar M , et al. Expression and cellular distribution of multidrug resistance‐related proteins in patients with focal cortical dysplasia. Seizure. 2007;16:493–503. 17482840 10.1016/j.seizure.2007.03.011

[44] Aronica E , Gorter JA , Jansen GH , van Veelen CWM , van Rijen PC , Leenstra S , et al. Expression and cellular distribution of multidrug transporter proteins in two major causes of medically intractable epilepsy: focal cortical dysplasia and glioneuronal tumors. Neuroscience. 2003;118:417–29. 12699778 10.1016/s0306-4522(02)00992-2

[45] Aronica E , Gorter JA , Jansen GH , van Veelen CW , van Rijen PC , Ramkema M , et al. Expression and cell distribution of group I and group II metabotropic glutamate receptor subtypes in Taylor‐type focal cortical dysplasia. Epilepsia. 2003;44:785–95. 12790891 10.1046/j.1528-1157.2003.54802.x

[46] Czornyj L , Lubieniecky F , Pomata H , Lazarowski A . Breast cancer resistant protein (BCRP) in cortical dysplasia with refractory epilepsy and failure to produce pharmacological coma. Epilepsia. 2005;46:326.

[47] Czornyj L , Lubieniecki F , Camarero S , Taratuto AL , Lazarowski A . BCRP expression with lipofuscin accumulation in abnormal neurons from a child with transmantle cortical dysplasia (TMCD) and refractory epilepsy. J Diagn Case Rep. 2021;2(3):1–5.

[48] Czornyj L , Lazarowski A . ABC‐transporters as stem‐cell markers in brain dysplasia/tumor epilepsies. Front Biosci (Landmark Ed). 2014;19:1425–35. 24896362 10.2741/4293

[49] Lazarowski A , Lubieniecki FC , Cuccia V , Taratuto A . Stem‐cell marker CD34, multidrug resistance proteins P‐gp and BCRP in SEGA. Receptors Clin Investig. 2014;1:e53. 10.14800/rci.53

[50] Volk HA , Burkhardt K , Potschka H , Chen J , Becker A , Löscher W . Neuronal expression of the drug efflux transporter P‐glycoprotein in the rat hippocampus after limbic seizures. Neuroscience. 2004;123(3):751–9. 14706787 10.1016/j.neuroscience.2003.10.012

[51] Aviles‐Reyes RX , Angelo MF , Villarreal A , Rios H , Lazarowski A , Ramos AJ . Intermittent hypoxia during sleep induces reactive gliosis and limited neuronal death in rats: implications for sleep apnea. J Neurochem. 2010;112:854–69. 20002528 10.1111/j.1471-4159.2009.06535.x

[52] Höcht C , Lazarowski A , Gonzalez NN , Auzmendi J , Opezzo JA , Bramuglia GF , et al. Nimodipine restores the altered hippocampal phenytoin pharmacokinetics in a refractory epileptic model. Neurosci Lett. 2007;413(2):168–72. 17240061 10.1016/j.neulet.2006.11.075

[53] Hoffman MM , Wei LY , Roepe PD . Are altered pHi and membrane potential in hu MDR1 transfectants sufficient to cause MDR protein‐mediated multidrug resistance? J Gen Physiol. 1996;108:295–313. 8894978 10.1085/jgp.108.4.295 PMC2229331

[54] Wadkins RM , Roepe PD . Biophysical aspect of P‐glycoprotein‐mediated multidrug resistance. Int Rev Cytol. 1997;171:121–65. 9066127 10.1016/s0074-7696(08)62587-5

[55] Auzmendi JA , Orozco‐Suárez S , Bañuelos‐Cabrera I , González‐Trujano ME , González EC , Rocha L , et al. P‐glycoprotein contributes to cell membrane depolarization of hippocampus and neocortex in a model of repetitive seizures induced by pentylenetetrazole in rats. Curr Pharm Des. 2013;19:6732–8. 23530507 10.2174/1381612811319380006

[56] Madison D , Niedermeyer E . Epileptic seizures resulting from acute cerebral anoxia. J Neurol Neurosurg Psychiatry. 1970;33:381–6. 5431726 10.1136/jnnp.33.3.381 PMC493484

[57] Semenza GL . A compendium of proteins that interact with HIF‐1alpha. Exp Cell Res. 2017;356(2):128–35. 10.1016/j.yexcr.2017.03.041 28336293 PMC5541399

[58] Merelli A , Caltana L , Girimonti P , Ramos AJ , Lazarowski A , Brusco A . Recovery of motor spontaneous activity after intranasal delivery of human recombinant erythropoietin in a focal brain hypoxia model induced by CoCl_2_ in rats. Neurotox Res. 2011;20:182–92. 21116766 10.1007/s12640-010-9233-8

[59] Bauer B , Hartz AMS , Pekcec A , Toellner K , Miller DS , Potschka H . Seizure‐induced up‐regulation of P‐glycoprotein at the blood‐brain barrier through glutamate and cyclooxygenase‐2 signaling. Mol Pharmacol. 2008;73:1444–53. 18094072 10.1124/mol.107.041210

[60] Comerford KM , Wallace TJ , Karhausen J , Louis NA , Montalto MC , Colgan SP . Hypoxia‐inducible factor‐1‐dependent regulation of the multidrug resistance (MDR1) gene. Cancer Res. 2002;62:3387–94. 12067980

[61] Lazarowski A , Caltana L , Merelli A , Rubio MD , Ramos AJ , Brusco A . Neuronal mdr‐1 gene expression after experimental focal hypoxia: a new obstacle for neuroprotection? J Neurol Sci. 2007;258(1–2):84–92. 17459414 10.1016/j.jns.2007.03.004

[62] Merelli A , Ramos AJ , Lazarowski A , Auzmendi J . Convulsive stress mimics brain hypoxia and promotes the P‐glycoprotein (P‐gp) and erythropoietin receptor overexpression. Recombinant human erythropoietin effect on P‐gp activity. Front Neurosci. 2019;13:750. 31379495 10.3389/fnins.2019.00750 PMC6652211

[63] Merelli A , Czornyj L , Lazarowski A . Erythropoietin: a neuroprotective agent in cerebral hypoxia, neurodegeneration, and epilepsy. Curr Pharm Des. 2013;19(38):6791–801. 23530506 10.2174/1381612811319380011

[64] Merelli A , Repetto M , Lazarowski A , Auzmendi J . Hypoxia, oxidative stress, and inflammation: three faces of neurodegenerative diseases. J Alzheimers Dis. 2021;82(s1):S109–26. 33325385 10.3233/JAD-201074

[65] Vega‐García A , Orozco‐Suárez S , Villa A , Rocha L , Feria‐Romero I , Alonso Vanegas MA , et al. Cortical expression of IL1‐beta, Bcl‐2, Caspase‐3 and 9, SEMA‐3a, NT‐3 and P‐glycoprotein as biological markers of intrinsic severity in drug‐resistant temporal lobe epilepsy. Brain Res. 2021;1758:14730. 10.1016/j.brainres.2021.147303 33516813

[66] Lorigados Pedre L , Morales Chacón LM , Orozco Suárez S , Pavón Fuentes N , Estupiñán Díaz B , Serrano Sánchez T , et al. Inflammatory mediators in epilepsy. Curr Pharm Des. 2013;19:6766–72. 23530510 10.2174/1381612811319380009

[67] Marchi N , Granata T , Ghosh C , Janigro D . Blood‐brain barrier dysfunction and epilepsy: pathophysiologic role and therapeutic approaches. Epilepsia. 2012;53:1877–86. 22905812 10.1111/j.1528-1167.2012.03637.x PMC4842020

[68] Janigro D . Blood‐brain barrier, ion homeostasis and epilepsy: possible implications towards the understanding of ketogenic diet mechanisms. Epilepsy Res. 1999;37:223–32. 10584972 10.1016/s0920-1211(99)00074-1

[69] Bazhanova ED , Kozlov AA , Litovchenko AV . Mechanisms of drug resistance in the pathogenesis of epilepsy: role of neuroinflammation. A literature review. Brain Sci. 2021;11:663. 34069567 10.3390/brainsci11050663 PMC8161227

[70] Zhang L , Ong WY , Lee T . Induction of P‐glycoprotein expression in astrocytes following intracerebroventricular kainate injection. Exp Brain Res. 1999;126(4):506–16. 10.1007/s002210050759 10422714

[71] Ramos AJ , Lazarowski A , Villar MJ , Brusco A . Transient expression of MDR‐1/P‐glycoprotein in a model of partial cortical devascularization. Cell Mol Neurobiol. 2004;24:101–7. 15049514 10.1023/B:CEMN.0000012728.19117.73 PMC11529942

[72] Shankar R , Donner EJ , McLean B , Nashef L , Tomson T . Sudden unexpected death in epilepsy (SUDEP): what every neurologist should know. Epileptic Disord. 2017;19(1):1–9. 28218059 10.1684/epd.2017.0891

[73] Bruno E , Maira G , Biondi A , Richardson MP ; RADAR‐CNS Consortium . Ictal hypoxemia: A systematic review and meta‐analysis. Seizure. 2018;63:7–13. 30391664 10.1016/j.seizure.2018.10.011

[74] Verrier RL , Pang TD , Nearing BD , Schachter SC . The epileptic heart: concept and clinical evidence. Epilepsy Behav. 2020;105:106946. 32109857 10.1016/j.yebeh.2020.106946

[75] Cordon‐Cardo C , O’Brien JP , Boccia J , Casals D , Bertino JR , Melamed MR . Expression of the multidrug resistance gene product (P‐glycoprotein) in human normal and tumor tissues. J Histochem Cytochem. 1990;38:1277–87. 1974900 10.1177/38.9.1974900

[76] Meissner K , Sperker B , Karsten C , zu Schwabedissen HM , Seeland U , Böhm M , et al. Expression and localization of P‐glycoprotein in human heart: effects of cardiomyopathy. J Histochem Cytochem. 2002;50:1351–6. 12364568 10.1177/002215540205001008

[77] Lazarowski AJ , García Rivello HJ , Vera Janavel GL , Cuniberti LA , Cabeza Meckert PM , Yannarelli GG , et al. Cardiomyocytes of chronically ischemic pig hearts express the MDR‐1 gene‐encoded P‐glycoprotein. J Histochem Cytochem. 2005;53:845–50. 15995143 10.1369/jhc.4A6542.2005

[78] Laguens RP , Lazarowski AJ , Cuniberti LA , Vera Janavel GL , Cabeza Meckert PM , Yannarelli GG , et al. Expression of the MDR‐1 gene‐encoded P‐glycoprotein in cardiomyocytes of conscious sheep undergoing acute myocardial ischemia followed by reperfusion. J Histochem Cytochem. 2007;55:191–7. 17101727 10.1369/jhc.6A7026.2006

[79] Auzmendi J , Salgueiro J , Canellas C , Zubillaga M , Men P , et al. Pilocarpine‐induced status epilepticus (SE) induces functional and histological P‐glycoprotein overexpression in cardiomyocytes, heart dysfunction and high ratio of sudden death in rats. In Proceedings of the Annual Meeting of American Epilepsy Society, Washington, DC, USA, 1–5 December 2017.

[80] Auzmendi J , Puchulu MB , Rodríguez GJC , Balaszczuk AM , Lazarowski A , Merelli A . EPO and EPO‐Receptor system as potential actionable mechanism for the protection of brain and heart in refractory epilepsy and SUDEP. Curr Pharm Des. 2020;26:1356–64. 32072891 10.2174/1381612826666200219095548

[81] Köhling R , Wolfart J . Potassium channels in epilepsy. Cold Spring Harb Perspect Med. 2016;6:a022871. 27141079 10.1101/cshperspect.a022871 PMC4852798

[82] Abraham MR , Janhangir A , Alekseev AE , Terzic A . Channelopathies of inwardly rectifying potassium channels. FASEB J. 1999;13:1901–10. 10544173 10.1096/fasebj.13.14.1901

[83] Akyüz E , Mega Tiber P , Beker M , Akbaş F . Expression of cardiac inwardly rectifying potassium channels in pentylenetetrazole kindling model of epilepsy in rats. Cell Mol Biol. 2018;64:47–54. 30672436

[84] Auzmendi JA , Lazarowski AJ . Seizures induce hypoxia and hypoxia induces seizures: a perverse relationship that increases the risk of SUDEP. Neurol Disord Epilepsy J. 2020;3(2):135.

[85] Friedman A . Blood‐brain barrier dysfunction, status epilepticus, seizures, and epilepsy: a puzzle of a chicken and egg? Epilepsia. 2011;52(Suppl 8):19–20. 21967353 10.1111/j.1528-1167.2011.03227.x PMC3234990

[86] Lazarowski A . P‐glycoprotein expression in heart, and its potential physiological relevance. J Cardiol Cardiovasc Ther. 2018;10(5):JOCCT.MS.ID.555799.

[87] Farrelly AM , Vlachou S , Grintzalis K . Efficacy of phytocannabinoids in epilepsy treatment: novel approaches and recent advances. Int J Environ Res Public Health. 2021;18:3993. 33920188 10.3390/ijerph18083993 PMC8070313

[88] Friedman D , Devinsky O . Cannabinoids in the treatment of epilepsy. N Engl J Med. 2015;373:1048–58. 26352816 10.1056/NEJMra1407304

[89] Rocha L , Frías‐Soria CL , Ortiz JG , Auzmendi J , Lazarowski A . Is cannabidiol a drug acting on unconventional targets to control drug‐resistant epilepsy? Epilepsia Open. 2020;5:36–49. 32140642 10.1002/epi4.12376 PMC7049809

[90] Auzmendi J , Palestro P , Blachman A , Gavernet L , Merelli A , Talevi A , et al. Cannabidiol (CBD) inhibited rhodamine‐123 efflux in cultured vascular endothelial cells and astrocytes under hypoxic conditions. Front Behav Neurosci. 2020;14:32. 32256321 10.3389/fnbeh.2020.00032 PMC7090129

[91] Lattanzi S , Trinka E , Striano P , Rocchi C , Salvemini S , Silvestrini M , et al. Highly purified cannabidiol for epilepsy treatment: a systematic review of epileptic conditions beyond Dravet syndrome and Lennox‐Gastaut syndrome. CNS Drugs. 2021;35:265–81. 33754312 10.1007/s40263-021-00807-y PMC8005394

[92] Lazarowski A , Czornyj L , Lubieniecki F , Vazquez S , D’Giano C , Sevlever G , et al. Multidrug‐resistance (MDR) proteins develop refractory epilepsy phenotype: clinical and experimental evidence. Curr Drug Ther. 2006;1(3):291–309.

[93] Lazarowski A , Czornyj L , Lubienieki F , Girardi E , Vazquez S , D'Giano C . ABC transporters during epilepsy and mechanisms underlying multidrug resistance in refractory epilepsy. Epilepsia. 2007;48(Suppl 5):140–9. 17910594 10.1111/j.1528-1167.2007.01302.x

